# Comprehensive analysis of circRNA expression profiles in rat cerebral cortex after moderate traumatic brain injury

**DOI:** 10.7150/ijms.71769

**Published:** 2022-04-18

**Authors:** Gang Li, Shaoping Li, Ruining Liu, Jiangtao Yu, Haoli Ma, Yan Zhao

**Affiliations:** 1 Emergency Center, Zhongnan Hospital of Wuhan University, Wuhan 430071, China.; 2 Department of Biological Repositories, Zhongnan Hospital of Wuhan University, Wuhan 430071, China.

**Keywords:** circular RNA, moderate traumatic brain injury, brain injury, gene expression profiles, rat

## Abstract

Traumatic brain injury is a medical event of global concern, and a growing body of research suggests that circular RNAs can play very important roles in traumatic brain injury. To explore the functions of more novel and valuable circular RNA in traumatic brain injury response, a moderate traumatic brain injury in rats was established and comprehensive analysis of circular RNA expression profiles in rat cerebral cortex was done. As a result, 301 up-regulated and 284 down-regulated circular RNAs were obtained in moderate traumatic brain injury rats, the Gene Ontology and Kyoto Encyclopedia of Genes and Genomes enrichment analysis were performed based on the circular RNA's host genes, and a circRNA-miRNA interaction network based on differentially expressed circular RNAs was constructed. Also, four circular RNAs were validated by RT-qPCR and Sanger sequencing. This study showed that differentially expressed circular RNAs existed between rat cerebral cortex after moderate traumatic brain injury and control. And this will provide valuable information for circular RNA research in the field of traumatic brain injury.

## Introduction

Traumatic brain injury (TBI) is a global public health problem, as an estimated about 50 million people will suffer this injury per year. It is a major cause of death and long-term disabilities in younger patients in developed countries 1,2. Based on severity, TBI ranges from a mild concussion to severe injury. In moderate to severe TBI, mortality is highest due to the primary injury, secondary insults, for instance, hypoxia or hypotension, and the development of associated complications, including those localized to the cranial vault and systemic problems associated with critical illness 3. As a complication, impaired cerebral autoregulation after moderate or severe TBI has been associated with a poor prognosis 4. However, there are currently no effective neuroprotective therapies that have been shown to alleviate TBI-induced injury 5. Therefore, translation of the many pathogenic mechanisms identified in animal studies to the development of treatment for human TBI remains a considerable challenge, especially in confirming whether the disrupted autoregulation attributes to the changes in specific molecular mediators.

Recently, accumulating evidence indicate that TBI significantly alters the expression of non-coding RNAs (ncRNAs), including long non-coding RNA (lncRNA) and microRNA (miRNA) 6, and neurological damage can be attenuated by normalizing the levels of certain lncRNAs and miRNAs 7-10. However, the biological functions of ncRNAs in TBI, especially for the circular RNAs (circRNAs), remain largely unknown and enigmatic. CircRNAs are a type of endogenous non-coding RNAs (ncRNAs) with complex stage and tissue-specific expression patterns 11, 12. Unlike linear RNA, circRNAs are much more stable and form a covalently closed-loop structure without 5'-3' polarity and a poly(A) tail, and might inhibit the function of miRNA as miRNA sponges to regulate the expression of host genes by the competing endogenous RNA (ceRNA) network 12-14. They are highly enriched in the brain and the changes in circRNA levels are thought to be associated with the development of diverse diseases, including stroke and neurodegenerative diseases 15-19. In the hippocampus of Aβ1-42-induced Alzheimer's disease-like rats, the circRNA-associated-ceRNA networks are reported as an important regulator of gene expression 20. Zhang et al. characterized the expression pattern of circRNAs and constructed the circRNA-associated-ceRNA networks in the cerebral cortex of senescence-accelerated prone 8 mice 19. It also has been shown that the change of circRNA expression pattern may be involved in physiological and pathological processes after traumatic spinal cord injury 21. All these findings further expanded our knowledge of circRNAs and contributed to understanding their regulation roles in brain diseases.

It has been reported that circRNAs are significantly altered in the hippocampus after TBI. Moreover, circRNAs may not only involve in brain damage but also neural regeneration following TBI through bioinformatics analysis and prediction of circRNA-miRNA interaction 22. The expression profile of circRNAs in exosomes, which come from the brain extracellular space, could be altered in mice after TBI. In addition, these differentially expressed circRNAs might be related to the growth and repair of neurons, the development of the nervous system, and so on 22. Many circRNAs are significantly changed in the traumatic cerebral penumbra cortex after TBI. For example, the circRNA chr8_87859283-87904548 potentially promotes neuroinflammation and impedes neurological restoration after TBI 23. In addition, some researches also show that altered circRNAs are mainly related to inflammation, cell death, repair of injury, and synaptic function, which were involved in the secondary cascade reaction of TBI. CircRNA_01564, circRNA_11926, circRNA_05015, circRNA_16282, and circRNA_05652 are found to play central roles in the crosstalk relationship, while circRNA_16895-miRNA myosin-10 is predicted to modulate fragment crystallizable gamma receptors (FcγR)-mediated phagocytosis pathway 24. It is also demonstrated that altered circRNA expression patterns might play important roles in post-TBI pathophysiological mechanisms 25.

However, the potential roles of circRNA in the physiological and pathological processes after TBI are still needed to be explored, especially in the role of circRNA-associated-ceRNA networks in the brain cortex after mTBI in rats.

In this study, to explore the target of effective therapeutic strategies for TBI, a high-throughput whole transcriptome sequencing was performed to identify differentially expressed profiles of circRNA in the cerebral cortex of moderate TBI (mTBI) model rats. Gene ontology (GO) and Kyoto Encyclopedia of Genes and Genomes (KEGG) pathway analyses were performed, and a circRNA-miRNA network was also constructed. Finally, four differentially expressed circRNAs were validated by RT-qPCR and Sanger sequencing. Our findings enriched our understanding of mTBI-associated circRNAs and provided significant evidence to further study the function of circRNAs in TBI response.

## Materials and methods

### Animals

Adult male Sprague-Dawley rats (weight 250-300g) were purchased from Vital River Laboratory Animal Technology Co. Ltd. (Beijing, China). The animal experiment was approved by the Animal Experiment Center and ethics committee of Zhongnan Hospital of Wuhan University and followed the National Institutes of Health Guide for the Care and Use of Laboratory Animals. Rats were housed for at least 7 days before establishing an animal model in a temperature (22-25 °C) and humidity-controlled (50% relative humidity) animal facility with a 12 h light/dark cycle. Animals had free access to food and water except that food was withheld overnight before surgery.

### Moderate traumatic brain injury model in rats

Eight rats were randomly divided into two groups of four rats each. As described previously in our previous study, animal models with moderate TBI (mTBI) were prepared with a weight-drop device 26. Briefly, the rats received 5% pentobarbital at a dose of 50 mg/kg by intraperitoneal injection. After anesthesia, the hair was shaved, and the skin was disinfected with iodophor. Rats were fixed on the brain stereotaxic device to cut the top of the skull skin and determine the bregma. Then a 5 mm diameter bone window was drilled to expose the dura mater. A 40 g hammer fell at 20 cm vertically along the outer tube leading to mTBI. For the sham-operated group, the animals underwent the same surgical procedure without weight-drop impact. 24 h later, the rats were analyzed for function performance and then were euthanized with sodium pentobarbital (100 mg/kg body weight) via intraperitoneal injection. Rats were housed in individual cages after surgery and placed on heat pads (37 °C) for 24 h to maintain normal body temperature during the recovery periods.

### Tissue collection, RNA isolation and sequencing

At 24h after mTBI, animals were anesthetized and transcardially perfused with 100 ml of 4 °C isotonic saline. The ipsilateral cortex around the injury site was dissected rapidly in the mTBI group and the cerebral cortex at the same site was obtained in the sham group. And then they were placed immediately into liquid nitrogen and stored at -80 °C.

Total RNA was isolated from eight ipsilateral cerebral cortex samples, including four mTBI samples and four control samples, using a miRNA Isolation Kit (Ambion, AM1560) following the manufacturer's protocol. The samples with RIN ≥ 7, monitored by the Agilent 2100 Bioanalyzer (Agilent Technologies, USA), were chosen for the following analysis. TruSeq Stranded Total RNA with Ribo-Zero Gold (Illumina, USA) was used for library construction, and the libraries were sequenced on the HiSeqTM 2500 platform.

### Data preprocessing and genomic alignment

Raw reads were quality filtered by Trimmomatic software (0.36), removing adapter and filtering out low-quality bases and N-bases or low-quality reads, and the high-quality clean reads were obtained 27. The FastaQC (v0.11.5) was used for quality control (https://www.bioinformatics.babraham.ac.uk/projects/fastqc/).

To generate the SAM file, we used BWA software (0.7.5a) to align the sequencing reads of each sample with the reference genome (Rnor_6.0, ftp://ftp.ncbi.nlm.nih.gov/genomes/all/GCF/000/001/895/GCF_000001895.5_Rnor_6.0/GCF_000001895.5_Rnor_6.0_genomic.fna.gz) 28.

### CircRNA prediction, differentially expressed circRNA analysis and clustering analysis

The circRNA identifier (CIRI) software (v2.0.3) was employed to scan for PCC signals (paired chiastic clipping signals), and circRNA sequences were predicted based on junction reads and GT-AG splicing signals 29.

For screening differential expression profiles of circRNAs, we used the estimateSizeFactors function of the DESeq R package to normalize the counts, and the nbinomTest function was used to calculate p-value and fold change values for the difference comparison 30. Differentially expressed transcripts with p-value ≤ 0.05 and fold change ≥ 2 or ≤ 0.5 were selected, and these differentially expressed circRNAs between control and TBI groups were identified, respectively. Hierarchical clustering was performed to show the distinguishable circRNAs expression pattern among samples. Moreover, the heatmap was constructed by using the pheatmap R package.

### GO annotations and KEGG pathway analyses, and circRNA-miRNA interaction research

For the differentially expressed circRNAs above, GO and KEGG enrichment analyses were done by Hypergeometric Distribution Test. The GO categories are derived from Gene Ontology (http://www.geneontology.org), which comprises three structured networks of defined terms that describe gene product attributes. Pathway analysis for differentially expressed circRNAs was performed, based on the latest KEGG (Kyoto Encyclopedia of Genes and Genomes; https://www.genome.jp/kegg) database, which allowed us to determine the biological pathways with the significantly enriched mRNAs. CircRNAs can serve as miRNA target molecules. And the miRanda software (v3.3a) was used to predict circRNA/miRNA interactions 31.

### Identification of miRNAs bound with the circRNAs and construction of circRNA-miRNA interaction network

Because circRNAs contain multiple miRNA binding sites, the circRNAs bound with miRNAs should be identified as the same as the way by which can predict miRNA targeted genes. In this study, miRanda (v3.3a) was used to identify the circRNAs binding with miRNAs. And the parameters were -sc 150 -en -30 -strict. A hypergeometric distribution test was used to screen the miRNAs which the differentially expressed circRNAs enriched. The circRNA-miRNA interaction network was constructed with Cytoscape 3.7.2.

### Real-time quantitative polymerase chain reaction (RT-qPCR) validation

RT-qPCR was used to confirm the differential expression identified in our RNA-seq. Before the reverse transcription reaction, the total RNA sample (≤ 1 µg) was digested with RNase-free DNase I for 30 min at 37 °C, which was stopped by adding 1µL EDTA (50 mM) at 65 °C for 10 min to inactivate DNase I. Then, the cDNA was synthesized from the digested total RNA using the PrimeScript RT reagent Kit (Takara Bio Company, Japan). The real-time qPCR reaction was performed using the ChamQ Universal SYBR qPCR Master Mix Q711 (Vazyme, China) in a QuantStudio 1 real-time PCR system (Thermofisher, USA) with the following conditions: 95 °C, 30 sec for one cycle; then 95 °C 10 sec and 60 °C 30 sec for 40 cycles. The specific quantitative primers were designed using Primer 3 (http://frodo.wi.mit.edu/primer3/). GAPDH was designed as an internal control. The 2^-ΔΔCt^ method was used to determine the relative quantification of gene expression levels. Each experiment had at least three replicates. The primers used in this study were listed in Table S3.

### Statistical analysis

All data were analyzed using GraphPad Prism 8 and presented as mean ± standard error of the mean (SEM). Student's t-tests were used for comparisons between two groups. False discovery rates (FDR) were calculated to correct p-values in RNA-seq analysis. Differences with p < 0.05 were considered to be statistically significant.

## Results

### Identification characterization of circRNAs in the rat cerebral cortex after mTBI

**Figure 1:**
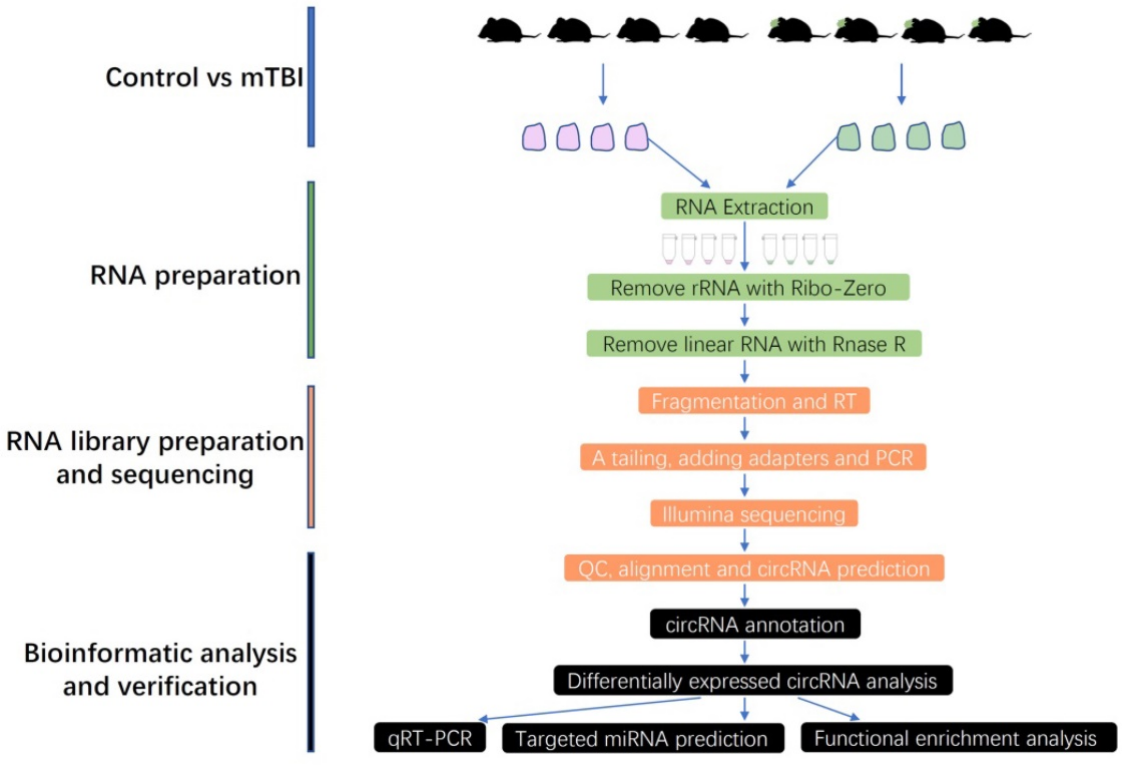
Workflow chart in this study. mTBI: Traumatic Brain Injury, RT: Reverse Transcription, QC: Quality Control, qRT-PCR: Quantitative Real Time Polymerase Chain Reaction.

To obtain more and precise circRNAs, the rRNA and linear RNAs were removed during RNA library preparation in this study, and the full workflow was shown in Figure 1. Finally, the circRNAs were systematically identified and annotated in the rat cerebral cortex after mTBI, and a total of 51995 circRNAs were identified from 2 groups including control and mTBI (Supplemental Table S1).

All the 51995 circRNAs can be classified into 2 main categories: Sense circRNA and antisense circRNA. By type and location, these two categories also can be divided into 4 subgroups respectively. For sense circRNAs, there were 96.12% genic_exonic, 1.87% genic_intronic, 1.06% intergenic_downstream, 0.95% intergenic_upstream, while for antisense circRNAs, the proportion were 30.27%, 11.54%, 15.16%, 43.03%, respectively (Supplemental Table S1, Supplemental Figure S1). The length of the circRNAs varied greatly. The longest circRNA was 99986 bp, the shortest one was only 45 bp, and about 63.39% cicrRNAs were shorter than 1000 bp (Supplemental Table S1). Moreover, about 70.00% cicrRNAs had 1 to 5 exons (Supplemental Figure S2), and most of circRNAs enriched at chromosomes 1 to 10 (Supplemental Figure S3). The amount of circRNA expression was estimated by RPM (reads per million) based on the number of back-spliced reads, and the distribution of circRNA expression was shown by a density distribution map (Supplemental Figure S4). This result showed that circRNAs generally had low expression levels.

### Differential expression of circRNAs in the rat cerebral cortex induced by mTBI

**Figure 2:**
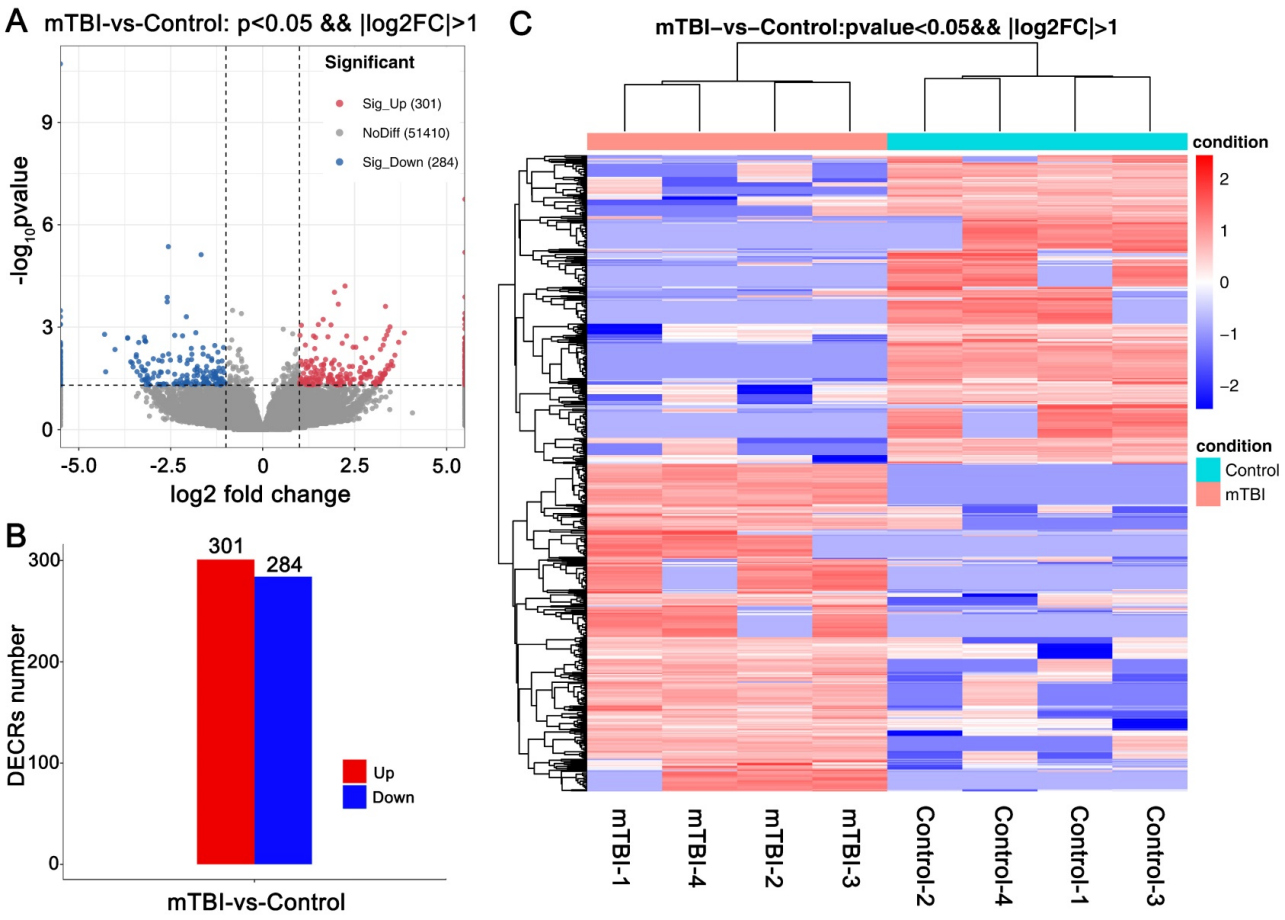
Differentially expressed circRNAs in rat cerebral cortex after mTBI. Volcano plot for DECRs in rat cerebral cortex after mTBI based on RNA_seq analysis. (B) Histogram plot for DECRs in mTBI compare to control. (C) Heatmap plot for DECRs. p < 0.05, |log2FC| > 1.

To clarify which circRNAs were essential for mTBI in the rat cerebral cortex. An analysis with differentially expressed circRNAs (DECRs) was carried out in this study. Finally, a total of 585 DECRs were identified and showed in the Volcano Plot based on a screening threshold p < 0.05, |log2FC| > 1 (Figure 2A, Supplemental Table S1), in which 301 up-regulated and 284 down-regulated circRNAs in mTBI rats compared to control (Figure 2B). There were 275 host genes in upregulated circRNAs, and 251 host genes in down-regulated circRNAs (Supplemental Table S1). The top 10 up-regulated and down-regulated circRNAs were listed in Table 1. Cluster analysis of DECRs based on the circRNAs expression was shown with a heatmap (Figure 2C). These DECRs may play important roles in the rat cerebral cortex induced by mTBI.

### GO and KEGG pathway enrichment analysis on the differentially expressed circRNAs

To learn about the related functions of DECRs, the GO and KEGG enrichment analyses were done based on the circRNA's host genes.

**Figure 3:**
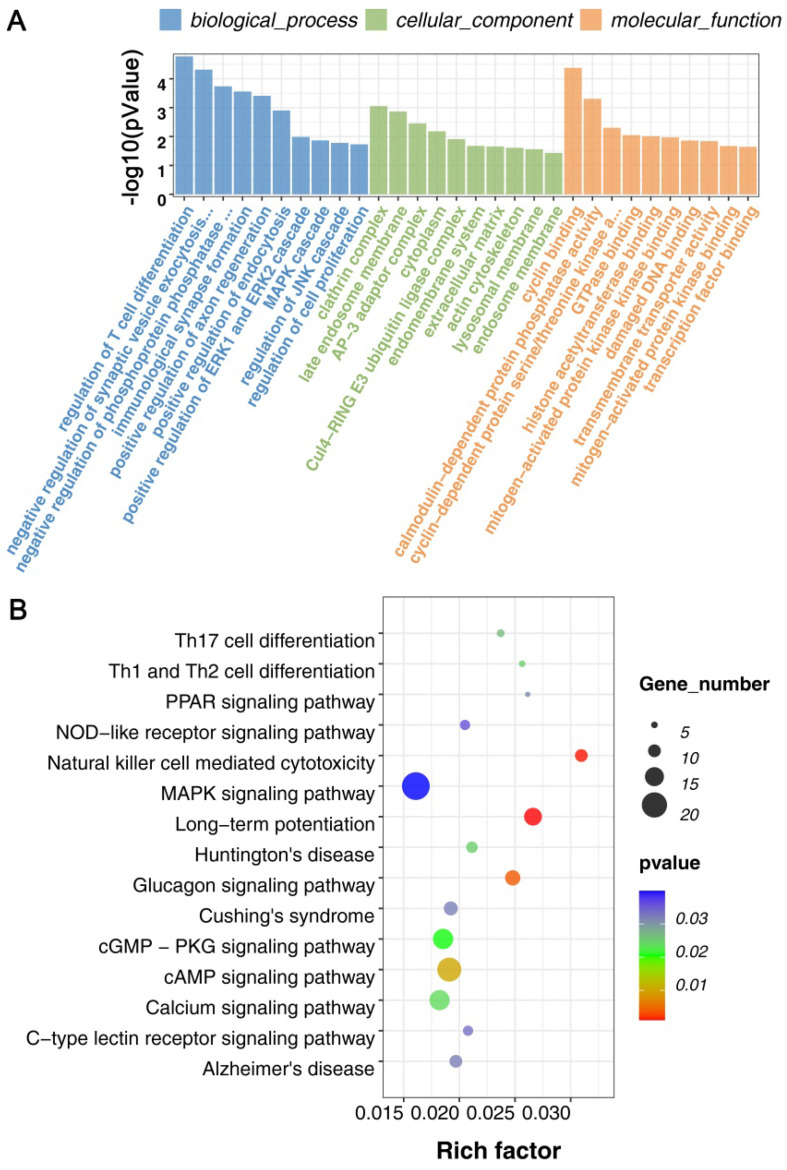
GO and KEGG enrichment analyses on DECRs in the rat cerebral cortex after mTBI. GO analysis on differentially expressed circRNAs (DECRs) in rat cerebral cortex after mTBI. (B) GO analysis on differentially expressed circRNAs (DECRs) in rat cerebral cortex after mTBI.

For all DECRs, there were 1281 GO terms were enriched (p < 0.05), including 890 GO BP (biological process), 132 GO CC (cellular component), 259 GO MF (molecular function). We further screened the terms including 3 genes at least and found that the DECRs were enriched in regulation of T cell differentiation, immunological synapse formation, positive regulation of axon regeneration, positive regulation of endocytosis and cell proliferation, and regulation of ERK1/ERK2, JNK, and MAPK cascade. (Figure 3A, Supplemental Table S1).

For KEGG pathway analysis, there were 47 KEGG pathways were enriched (p < 0.5) in all DECRs. And found that Natural killer cell mediated cytotoxicity, Th1, Th2, and Th17 cell differentiation, C-type lectin receptor signaling pathway, NOD-like receptor signaling pathway, MAPK signaling pathway, and some neurodegenerative disease pathway, for example, Huntington's disease and Alzheimer's disease, were enriched in the DECRs (Figure 3B, Supplemental Table S1).

These results suggested that cell proliferation and differentiation, immune response, and kinase-induced signal transduction may play essential roles in the mTBI response in the rat cerebral cortex.

### Prediction of circRNA-miRNA interaction network

**Figure 4:**
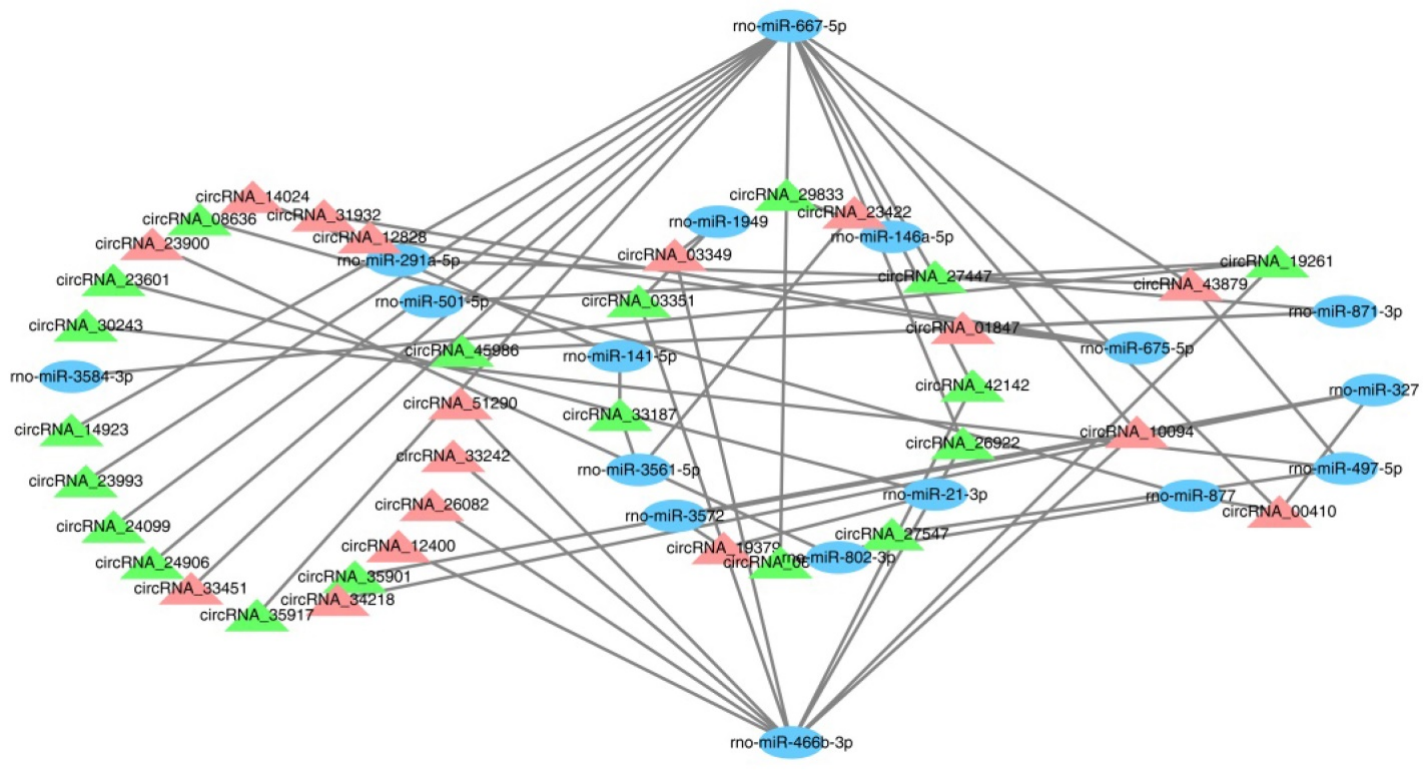
CircRNA-miRNA interaction network. The blue ovals show the targeted miRNAs, the green triangles show down-regulated circRNAs and the light red triangles show up-regulated circRNAs.

CircRNAs, as sponges for miRNAs, have been reported to indirectly modulate the expression level of other related RNAs by miRNA response elements 32. Therefore, it is very important to identify the interaction of circRNAs and miRNAs. In this study, 576 targeted miRNAs were identified for the DECRs (Supplemental Table S2). To understand which miRNAs were more effective for the DECRs, an enrichment analysis was done for all the miRNAs by a hypergeometric distribution test. As a result, the DECRs were significantly enriched in 49 miRNAs (p < 0.05) (Supplemental Table S2). 36 circRNAs and 17 miRNAs were selected to construct a circRNA-miRNA network (Figure 4, Supplemental Table S2). The result showed rno-miR-667-5p and rno-miR-466b-3p were regulated by a greater number of circRNAs (Figure 4). This result suggested rno-miR-667-5p and rno-miR-466b-3p may be participated in the mTBI response by interacting with these circRNAs.

### Validation of differentially expressed circRNAs by RT-qPCR

**Figure 5:**
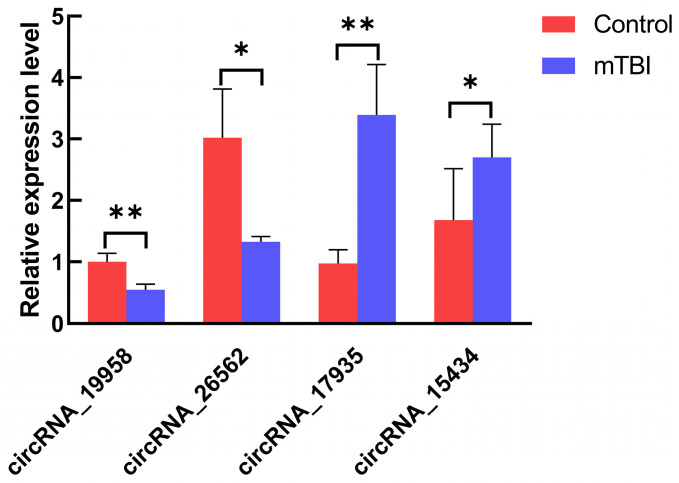
Expression level analysis on circRNAs by RT-qPCR. The expression of circRNA_19958, circRNA_26562, circRNA_17935 and circRNA_15434 were tested in at least 3 control s and 3 mTBI rat cerebral cortex samples by RT-qPCR. *: P < 0.05, **: P < 0.01.

To validate the transcription data, 10 genes were randomly chosen to test the expression level by the RT-qPCR using divergent primers. In our result, we found that four circRNAs were differentially expressed in the mTBI sample compared to the control. CircRNA_19958 and circRNA_26562 were down-regulated, while circRNA_15434 and circRNA_17935 were up-regulated significantly (Figure 5, Table 2). However, there are still 6 circRNAs were not significantly changed in expression level (Supplemental Figure S3).

**Figure 6:**
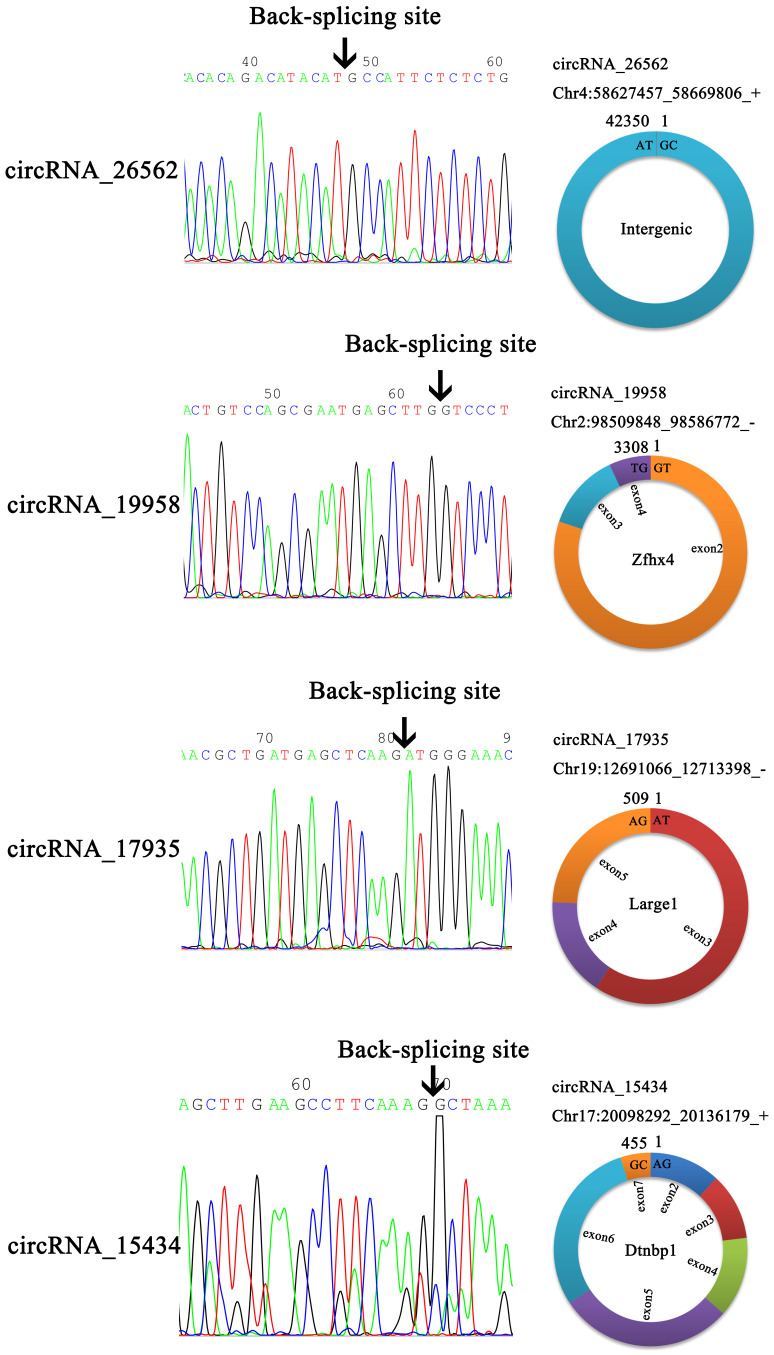
Head-to-tail splicing in the RT-qPCR product of circRNA.

In order to verify the back-splicing site and make clear the structure of the four circRNAs, the RT-qPCR products were used for Sanger sequencing. The results showed circ_19958, circ_17935, and circ_15434 were originated from a different number of exons, although circ_26562 was originated from an intergenic sequence (Figure 6).

## Discussion

As the body's most vulnerable organ, the brain is the center of human consciousness, controlling how we act, feel, speak, and so on 33-35. TBI is an important cause of morbidity and mortality worldwide. Many drug therapies have been developed to protect the brain after injury, however, none of those are successful in TBI outcomes 36-38. Therefore, TBI will bring heavy burdens to the patient's family. At present, a large number of studies have shown that circRNAs are very important in brain diseases such as Alzheimer's disease, Parkinson's disease, and glioma 39,40. It also has been reported that circRNA expression profiles of the TBI brain are different from that of the normal brain in both intracellular and extracellular space, suggesting that circRNAs may be involved in the pathogenesis of TBI and act as a regulator 22-24,41,42. As a result, exploring the novel circRNAs and making clear their function in recovering brain injuries have great significance. In this study, we predicted many novel and meaningful circRNAs in the rat mTBI model by high-throughput sequencing and verified 4 circRNAs by RT-qPCR and Sanger sequencing. This will provide new knowledge of circRNAs' functions on brain injuries in rats.

CircRNAs can regulate gene expression in different ways. Firstly, circRNAs can affect parental gene expression. This is mainly because the formation of circRNAs can influence the typical splicing of their precursor transcripts, leading to changes in gene expression levels 43. Some nuclear-located circRNAs may even regulate gene expression at the transitional and splicing levels, including CircSEP3 from exon 6 of SEPALLATA3, which can regulate the splicing of its linear counterpart 44,45. Secondly, circRNAs can be translated due to internal ribosome entry sites or N6-methyladenosine modification enriched in circRNAs 45,46. Besides, circRNAs also can act as a sponge for miRNAs to regulate gene expression indirectly. It's reported that as a sponge for miR-138, circRNA sex-determining region Y can prevent miR-138 from interacting with its target genes 13. To know the functions of predicted circRNAs in this study, we identified the target miRNAs and constructed a network between circRNA and miRNA. The network showed that rno-miR-466b-3p and rno-miR-667-5p may serve as a potential target of multiple circRNAs and be involved in their expression regulation. However, their relationship and downstream genes still needed to be confirmed and explored by further studies.

CircRNAs also play important roles in brain injury. A wide variety of studies have shown that circRNAs are enriched in brain tissues, which include the cortex, cerebellum, striatum, hippocampus, and olfactory bulbs, and are closely connected with neuronal development such as the development of neural stem cells 47-50. Besides the functions on neuronal development, they are also complexly linked to brain disorders, for example, Alzheimer's disease and temporal lobe epilepsy 51-53. Studies have shown that stroke-induced brain damage is mediated by multiple synergistic pathophysiologic mechanisms, including autophagy, mitochondrial dysfunction, apoptosis, inflammation, and so on 54,55. KEGG pathway enrichment analysis revealed that the cell cycle, mitogen-activated protein kinase (MAPK) signaling, focal adhesion, and regulation of the actin cytoskeleton are core pathways associated with circRNAs 15. In brain ischemia-reperfusion injury, apoptosis-related, immune-related, and metabolism-related pathways may have critical roles 56. However, in traumatic brain injury, it's also found that circRNAs can participate in the immune response, inflammation response, and neuronal apoptosis 24, 57-60. In our study, the GO analysis results showed differentially expressed circRNAs in the mTBI group were enriched immunological synapse formation, positive regulation of axon regeneration, positive regulation of ERK1 and ERK2 cascade, MAPK cascade, regulation of JNK cascade. On the other hand, the KEGG results showed that immune-related pathways such as NOD-like receptor signaling pathway, Natural killer cell mediated cytotoxicity, C-type lectin receptor signaling pathway, and Th1/2/17 cell differentiation, and some neurorelated diseases, Huntington's disease and Alzheimer's disease were also enriched in mTBI. As a result, circRNAs may participate in mTBI response regulation via regulating immune, inflammation, and apoptosis and it's very necessary for further exploring the molecular mechanisms.

## Conclusion

In conclusion, we report a circRNA expression profile in the rat cerebral cortex after moderate traumatic brain injury. In our study, 585 differentially expressed circular RNAs (DECRs) were identified, and the functional enrichment analysis was performed which may reveal the potential and important roles of circular RNAs in mTBI regulation. Besides that, A network between the DECRs and their target miRNAs was constructed and found that rno-miR-667-5p and rno-miR-466-3p may have an important role in the regulation of circular RNAs expression in mTBI response. Our study also confirmed four circRNAs expression levels between sham and mTBI, and further make clear their sequence composition. We believe that this study will bring valuable information to researchers in this field.

## Supplementary Material

Supplementary figures and table 3.

Supplementary table 1: Annotation of circRNA and DECRs.

Supplementary table 2: Interaction between circRNA and miRNA.

## Figures and Tables

**Table 1:** The characterization of top 10 up-regulated and top 10 down-regulated circRNAs

circRNA_id	log2FC	pval	up-down	circRNA_chr	circRNA_strand	circRNA_start	circRNA_end	genomic_length	circRNA_length	Type	gene
circRNA_04099	Inf	1.90E-16	Up	Chr1	+	278661823	278749829	88007	758	sense-overlapping	Atrnl1
circRNA_35079	Inf	1.22E-08	Up	Chr7	-	98606921	98682856	75936	63953	sense-overlapping	Tmem65
circRNA_18531	Inf	3.38E-06	Up	Chr19	+	46552916	46559348	6433	346	sense-overlapping	Vat1l
circRNA_03118	5.22	9.08E-06	Up	Chr1	-	221185801	221186464	664	664	intergenic	---
circRNA_15598	2.47	1.78E-05	Up	Chr17	+	36602134	36627167	25034	267	sense-overlapping	Cdkal1
circRNA_10508	2.96	5.83E-05	Up	Chr13	+	101274882	101280792	5911	363	sense-overlapping	Susd4
circRNA_17935	2.65	2.84E-04	Up	Chr19	-	12691066	12713398	22333	509	sense-overlapping	Large1
circRNA_27089	11.94	4.62E-04	Up	Chr4	+	97812913	97830459	17547	17547	intergenic	---
circRNA_15434	2.88	6.74E-04	Up	Chr17	+	20098292	20136179	37888	455	sense-overlapping	Dtnbp1
circRNA_40399	11.22	8.63E-04	Up	Chr9	-	119615345	119617261	1917	1917	exonic	Emilin2
circRNA_15695	0.27	2.04E-06	Down	Chr17	-	48657102	48679275	22174	793	sense-overlapping	Vps41
circRNA_33112	-lnf	2.56E-06	Down	Chr6	+	112684486	112689839	5354	823	sense-overlapping	Nrxn3
circRNA_22458	0.25	3.64E-04	Down	Chr20	-	31723858	31736111	12254	11458	sense-overlapping	RGD1305587
circRNA_24421	0.08	4.11E-04	Down	Chr3	+	104889557	104936645	47089	356	sense-overlapping	Fmn1
circRNA_13425	0.21	7.07E-04	Down	Chr15	+	61667133	61683055	15923	809	sense-overlapping	Mtrf1
circRNA_27617	0.18	7.58E-04	Down	Chr4	+	140372957	140379514	6558	576	sense-overlapping	Itpr1
circRNA_26650	0.39	1.82E-03	Down	Chr4	-	64265934	64270334	4401	453	sense-overlapping	Ptn
circRNA_19958	0.42	2.13E-03	Down	Chr2	-	98509848	98586772	76925	3386	sense-overlapping	Zfhx4
circRNA_13422	0.37	2.48E-03	Down	Chr15	+	61665894	61683055	17162	1235	sense-overlapping	Mtrf1
circRNA_21502	0.22	2.60E-03	Down	Chr2	+	217971903	217983563	11661	523	sense-overlapping	Olfm3

**Table 2:** The characterization of circRNAs verified by RT-qPCR

circRNA_id	log2FC	pval	up_down	circRNA_chr	circRNA_strand	circRNA_start	circRNA_end	genomic_length	circRNA_length	Type	gene
circRNA_17935	1.40	0.00028	Up	Chr19	-	12691066	12713398	22333	509	sense-overlapping	Large1
circRNA_15434	1.52	0.00067	Up	Chr17	+	20098292	20136179	37888	455	sense-overlapping	Dtnbp1
circRNA_19958	-1.24	0.00217	Down	Chr2	-	98509848	98586772	76925	3386	sense-overlapping	Zfhx4
circRNA_26562	-2.24	0.00347	Down	Chr4	+	58627457	58669806	42350	42350	intergenic	-
