# Supplementary material for: Comprehensive analysis of circRNA expression profiles in rat cerebral cortex after moderate traumatic brain injury
Source: Int J Med Sci. 2022 Apr 18;19(4):779–88. doi: 10.7150/ijms.71769 (PMC9108397; doi:10.7150/ijms.71769)
Supplement: Supplementary file 1 — Supplementary figures and table 3. [file ijmsv19p0779s1.pdf]

Supplementary files：

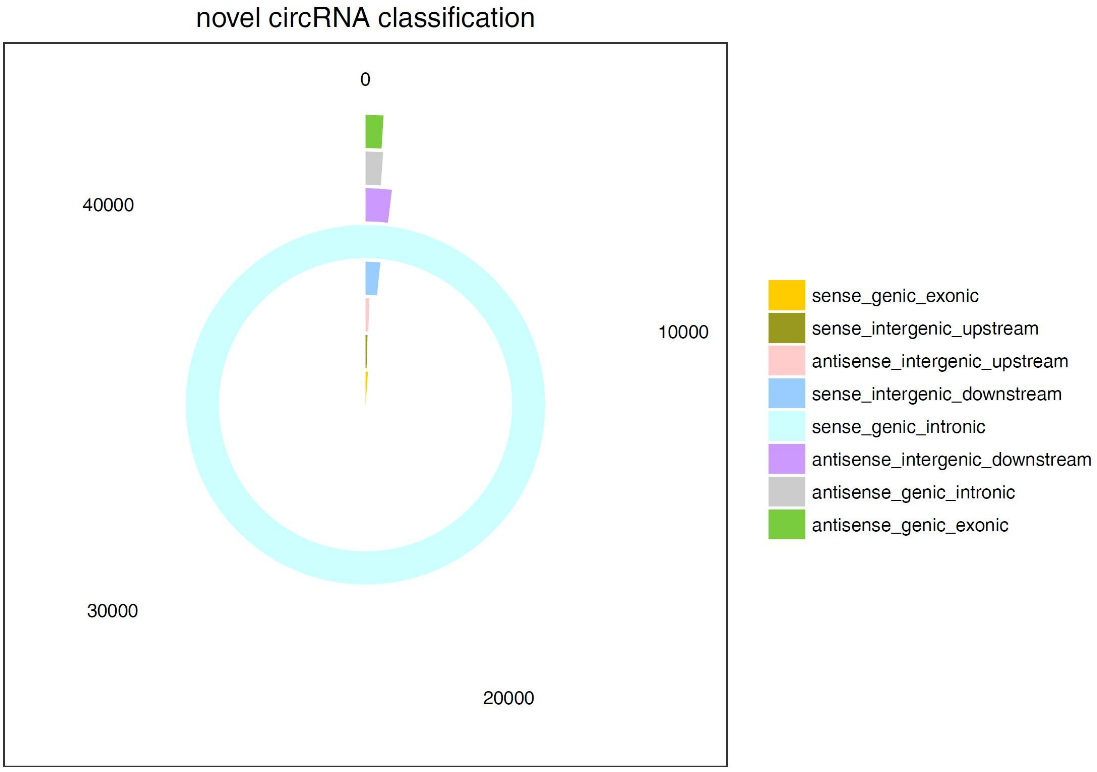

Figure S1 Classification of circRNAs identified in rat cerebral cortex after TBI.

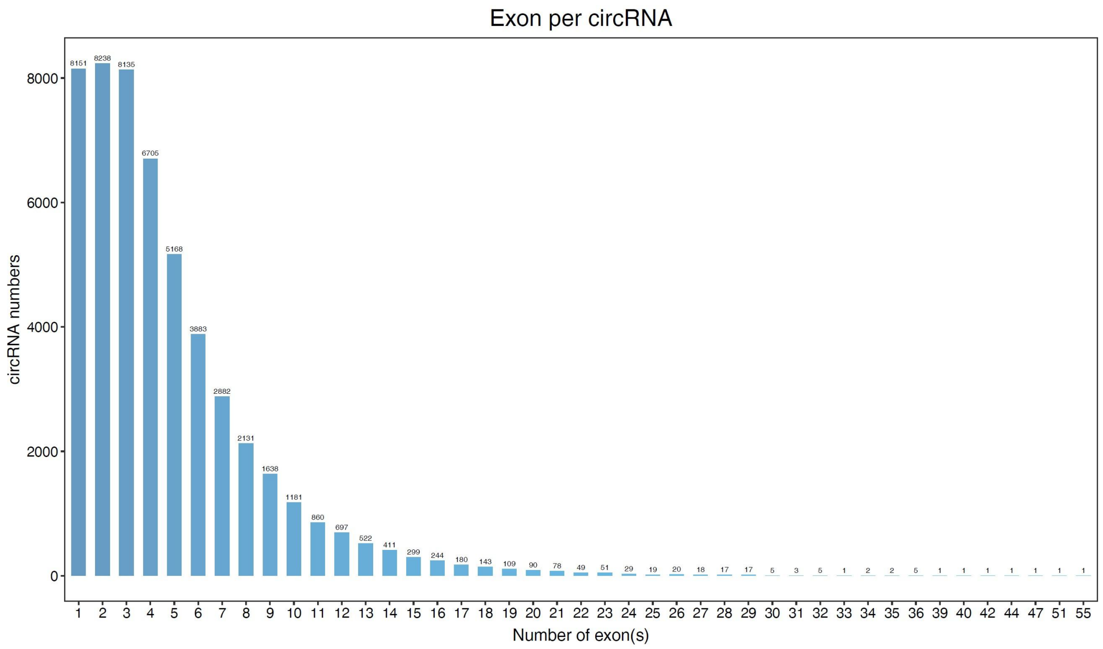

Figure S2 The exon number distribution of circRNAs.

Abscissa axis shows the number of exon(s), and the ordinate axis shows the circRNAs numbers.

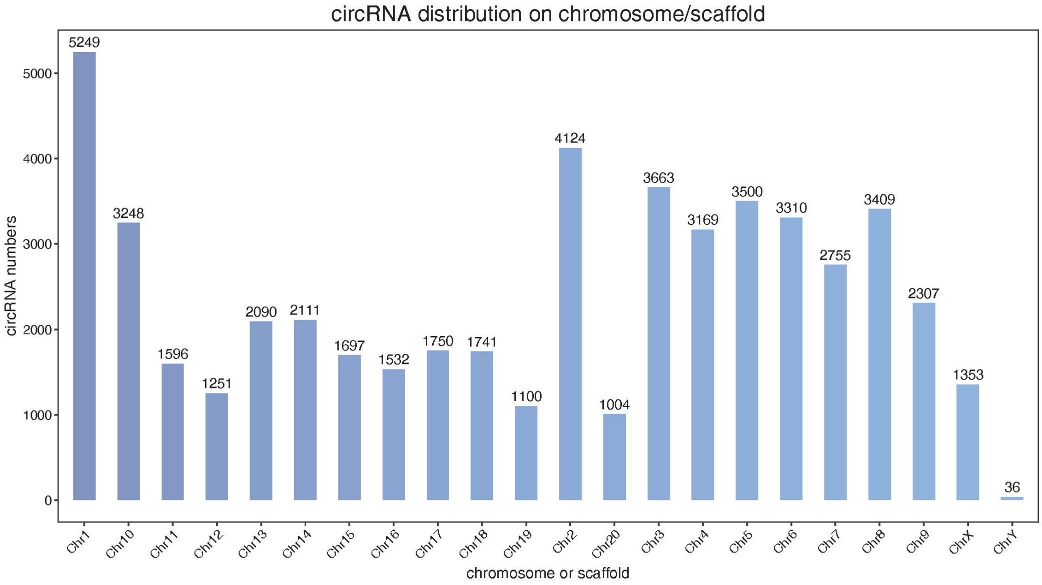

Figure S3 Distribution of circRNA on chromosome.

Abscissa axis shows the chromosome number, and the ordinate axis shows the circRNAs numbers.

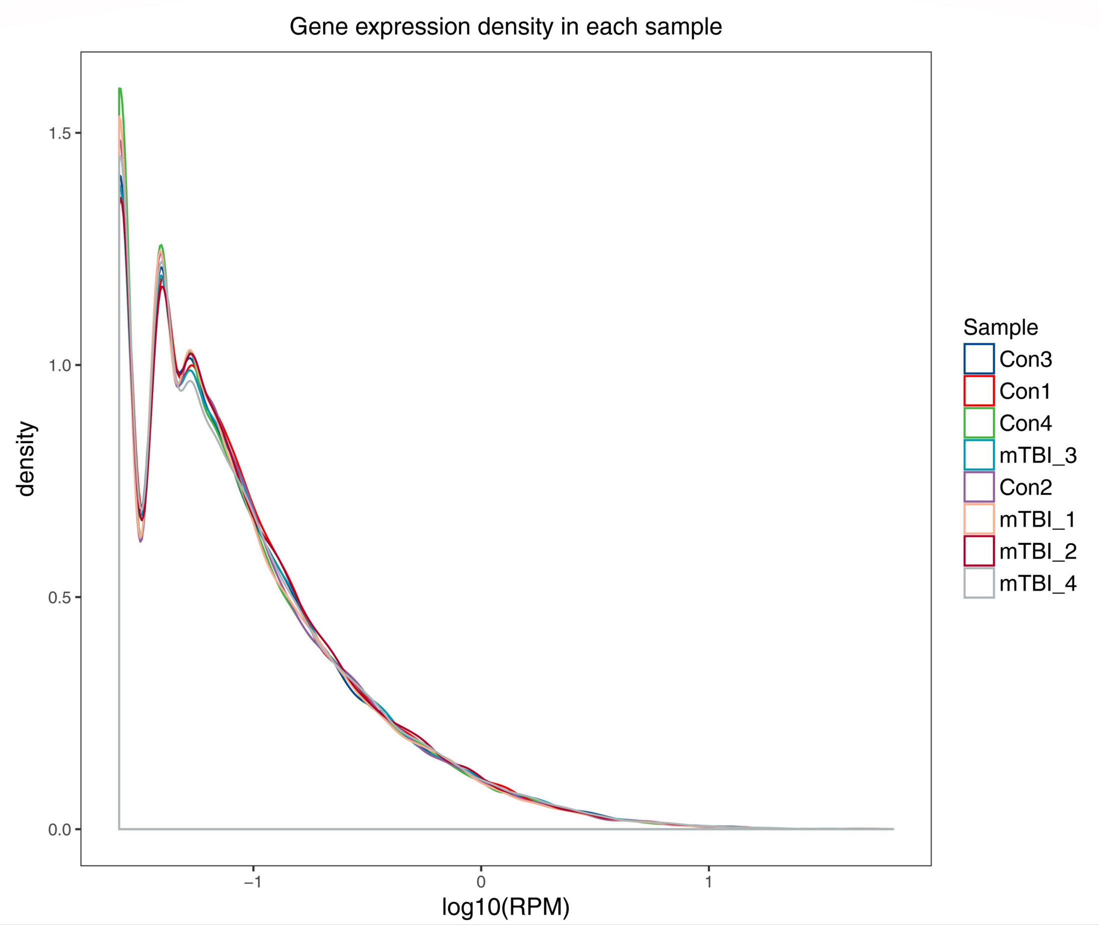

Figure S4 Distribution of RPM value.

Different lines with special color show different samples. Abscissa axis shows the log10(RPM) value, and the ordinate axis shows the probability density.

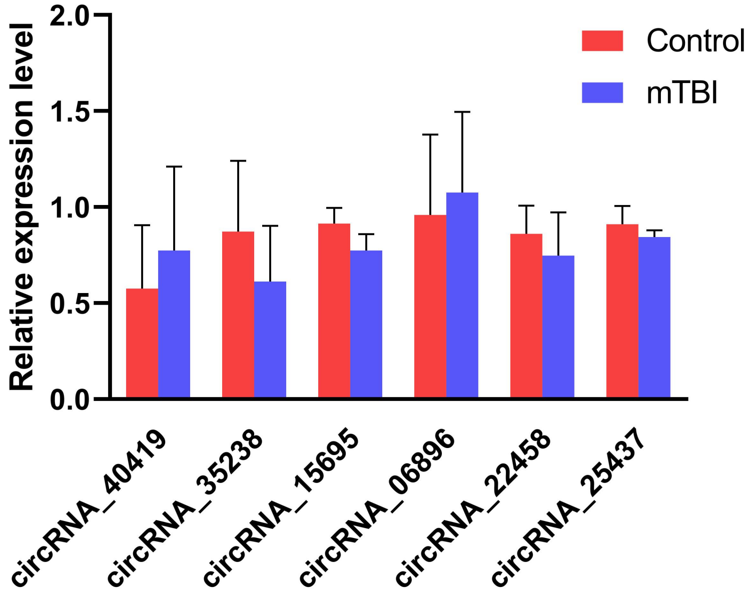

Figure S5 Expression level analysis on circRNAs by RT-qPCR.

The expression of circRNA_40419, circRNA_35238, circRNA_15695, circRNA_06896, circRNA_22458 and circRNA_25437 were tested in at least 3 control s and 3 mTBI rat cerebral cortex samples by RT-qPCR.

Table S3 The primers used in this study

primer		sequence
circRNA_19958	FP	5' ctccagctacaccagcaagg 3'
	RP	5' caattgctacatggggatctc 3'
circRNA_26562	FP	5' cacctacatggcagctcaca 3'
	RP	5' tcaggtaatcttggcagcatc 3'
circRNA_17935	FP	5' tccacctcattgctgattctatt 3'
	RP	5' tgggcttgggactccaggggagaga 3'
circRNA_15434	FP	5' cttgtgtgggcagtgtgagt 3'
	RP	5' cctgggtctgctttttacttt 3'
circRNA_40419	FP	5' atgaagatgacgatgatga 3'
	RP	5' atttcgagaagtagctgac 3'
circRNA_35238	FP	5' gagtatgactatgacgatgggta 3'
	RP	5' agtagggagattatggaggaa 3'
circRNA_15695	FP	5' caaggaacaagatgatgga 3'
	RP	5' gtggtttcagaacggggtc 3'
circRNA_06896	FP	5' tccgagaataaaacacagtgg 3'
	RP	5' aggcaaaaggatcagaagttc 3'
circRNA_22458	FP	5' aaagtcaggtgggaggaagc 3'
	RP	5' cctgaacgatctccccattg 3'
circRNA_25437	FP	5' ggaaagtaacatctcagacca 3'
	RP	5' gcaataaggtgaggaggtag 3'
GAPDH	FP	5' ctcatgaccacagtccatgc 3'
	RP	5' ttcagctctgggatgacctt 3'
